# Attempted suicide and pregnancy

**DOI:** 10.5249/jivr.v3i1.77

**Published:** 2011-01

**Authors:** Andrew E. Czeizel

**Affiliations:** ^a^Foundation for the Community Control of Hereditary Diseases, Budapest, Hungary.

## Abstract

**Background:**

The aim of the Budapest Monitoring System of Self-Poisoning Pregnant Women was to evaluate the potential congenital abnormality inducing effect of extremely large doses of drugs among pregnant women who attempted suicide. This system was appropriate to describe the characteristics of these pregnant women as a secondary finding from this model.

**Methods:**

All self-poisoned patients were cared for at a toxicological inpatient clinic in Budapest, between 1960 and 1993. Of a total of 1,044 pregnant women identified from the three different periods of the project, only 19 (1.8%) died. Women who survived were visited at home to reveal birth outcomes, and their exposed children were examined medically to identify congenital abnormalities and tested to estimate their cognitive-behavioral status. The previous or subsequent children of these pregnant women were used as controls with a similar examination protocol.

**Results:**

In general, self-poisoned pregnant women were young (peak age was between 18 and 20 years), 62% had their first pregnancy, 55% were unmarried, they had lower socioeconomic status, 46% were smokers and 22.5% drinkers, but depression/panic disorder occurred only among 17 pregnant women. Suicide attempts with drugs were most frequent in the fourth post-conceptional week and second month of pregnancy. In general they used smaller doses of drugs for suicide than non-pregnant age-matched women. Of 1,044 self-poisoned pregnant women, 926 had known pregnancy outcomes and 411 (44.4%) delivered live-born babies.

**Conclusions:**

The self-poisoning model appears to have several benefits (e.g., dose-response estimation of drugs) in comparison with other methods when evaluating teratogenic/fetotoxic effect of drugs. It is suggested that an international monitoring system of self-poisoned pregnant women should be established to provide a larger data base.

## Introduction

Suicide and self-inflicted injury are specified as intentional causes of death or diseases. However, suicide and attempted suicide are differentiated. Most suicide attempts are made among young people, mainly females who are otherwise healthy. In contrast, most suicide deaths occur in advanced age, in males, and in persons among whom psychiatric problems are often present.^1-3^

Suicide attempts have increased significantly during the 20th century, reaching annual registered suicide attempt rates of 0.38% in Boston in 1964-1992^4^and 0.23% in Budapest in the 1970s.^5^The proportion of suicide attempts made using drugs and other chemicals has also shown a dramatic rise (90%).^6-8^ This type of suicide has been termed self-poisoning.^9^The so-called self-poisoning epidemic has produced a major socio-medical problem, mostly among young women,^10-15^thus it may occur among pregnant women as well. The estimated rate of self-poisonings within the reproductive age may be about 6 %, i.e. 1 in 17 persons.^5^

Pregnancy is a special risk factor for suicide attempts among females. The incidence,^16^mortality,^17^demographic characteristics^18,19^and motivation^20,21^of suicide in pregnant women have been previously studied. Fortunately, the number of survivors after self-poisoning during pregnancy has increased significantly as the result of more effective medical care (e.g. ambulances) and treatment (in inpatient clinics).^5^Thus, the long term effects of self-poisoning, including the potential teratogenic/fetotoxic effects of drugs have become an important medical concern.

A teratogenic agent acts during pregnancy to produce a physical defect in the conceptus or offspring, mainly in the early phase of fetal development. A fetotoxic agent produces functional defects in the fetuses or newborn infants, mainly in the second part of pregnancy. The prediction of the teratogenic risk of drugs among humans is a difficult task due to methodological problems. The results of experimental animal investigations cannot be extrapolated for human fetuses due to species differences. Case reports and clinical series, in addition to analytical (e.g. case-control) epidemiological studies can predict the teratogenic and fetotoxic risk of drugs among humans but these predictions must be regarded with caution because of selection and recall bias, and usually have no appropriate controls and the lack of dose-response estimation. The clinical trials of a drug under development in general do not include pregnant women.^22^However, pregnant women, who survive suicide attempts by taking large doses of drugs may represent a unique model for studying human teratogenicity (ie. congenital abnormality inducing potential) and fetotoxicity of drugs and other chemical compounds.^5^

Thus we decided to use the socalled self-poisoning pregnant women model as a more effective approach to evaluate the potential teratogenicity/fetotoxicity of human exposure to drugs beyond experimental, clinical and analytical epidemiological studies. This approach was previously identified as “experimental” epidemiology,^23^although this term does not appear appropriate for studies of humans, therefore we suggest the term disaster epidemiology. Disaster epidemiology can apply to extraordinary natural catastrophes, e.g., earthquakes and human-made disasters such Hiroshima/ Nagasaki nuclear bomb attacks,^24^Chernobyl nuclear plant accident,^25,26^ accidents in the chemical industry, such in Seveso or Bophal, ^27^which offer a special opportunity to study the association between disorders, e.g., structural birth defects, i.e. congenital abnormalities, and specific environmental agents. Self-poisonings can be classified as continuous and not rare events in the human-made disaster epidemiology.

First the Monitoring System of Self-poisoning Pregnant Women within the Budapest Registry of Self-Poisoned Patients^3^is shown shortly. Previously the results of this project regarding the teratogenic/fetotoxic effect of drugs used frequently for suicide attempts were published,^28^here the maternal characteristics of pregnant women who attempted suicide are summarized.

**Self-poisoning pregnant women model**

The monitoring of self-poisoning pregnant women project was based on pregnant women who attempted suicide by means of drugs and were admitted to the Department of Toxicological Internal Medicine, Korányi Hospital, Budapest ^3^from the population of about 3 million people in Budapest, the capital of Hungary and the surrounding area. Pregnant women were identified among self-poisoned females in order to determine the teratogenic and fetotoxic effect of large doses of drugs on their exposed children.

Our project included three time periods, each with a different method of case ascertainment, and the optimal approach was achieved in the third period.

I.Self-poisoned pregnant women were ascertained by the review of admission and medical files of female patients in 1980 admitted to the study hospital between 1960 and 1979.^29,30^

II.Self-poisoned pregnant women were ascertained by a psychologist who visited the study hospital every second day and identified pregnant women through personal interviews based on case histories and missed menstrual period; these pregnancies were then confirmed by a gynecologist also between 1980 and 1984. ^31^

III.Self-poisoned pregnant women were ascertained by a sensitive pregnancy test in the blood samples (taken at their hospital admission for the measurement of suicidal drug doses) of all admitted women from 15 years to 50 years between 1985 and 1993.^32^

Gestational age is calculated from the first day of the last menstrual period; however, we used the concept of post-conceptional pregnancy age, which is estimated from the first day of the third week of the first lunar (28 day) pregnancy month. Thus the expected duration of pregnancy was 266 days and 38 weeks.

The doses and effects of drugs used for self-poisoning were estimated on the basis of data from three sources: (i) information obtained from the pregnant women, (ii) drug levels in their blood and (iii) the clinical pictures of intoxication. All pregnant women who survived their suicide attempt were visited later at home and their pregnancy outcomes were elucidated. Live-born exposed children and their siblings, of course with their mothers, were invited to our institute for a thorough medical and psychological examination. About 15 % of women with their children did not visit our institute; they were visited at home and examined on the basis of the study protocol of the project. The main focus of the study was structural birth defects, i.e. congenital abnormalities, though minor anomalies (such as unusual morphologic variants that are of no serious medical or cosmetic consequences) and cognitive/behavioral status were also examined in the exposed children and their siblings.

Sibling controls were selected from the previously and subsequently born unexposed child(ren) of self-poisoned pregnant women. If siblings were born to pregnant women who attempted suicide in their other pregnancies, they were excluded from unexposed sibling controls.

The strength of our study is the personal examination of exposed children and their sibling controls by the same methods and experts. Thus all congenital abnormalities were diagnosed by the well-defined diagnostic criteria in both exposed children and their siblings. In addition the exposure data (drugs, doses, time) were based on multiple sources of information. The major weakness of the project is connected with the different ascertainment of self-poisoned pregnant women in the three periods of the project.

The total material included 1,044 pregnant women, however, 19 pregnant women died due to suicide. Fortunately, the number of survivors after suicide during pregnancy has increased significantly during the study period between 1960 and 1993(Table 1). The rate of lethal suicide attempts in pregnant women was 6.7%, 1.1% and 0.4% in the first, second and third period of the project, respectively. Pregnant women with lethal outcome after suicide were excluded from the further study because we had no chance for the evaluation of pregnancy outcomes. A further 99 pregnant women had given a false address or they had a new, unknown residence, therefore their pregnancy outcomes could not be indentified(Table 1).Thus finally the pregnancy outcomes were evaluated in 926 women.

Further details of the evaluation methods and the results of the study were presented in more depth in a previous description of the Hungarian self-poisoning pregnant model.^28^

**Table 1:Number and rate (%) of pregnant women with lethal suicide events, the proportion of dropouts and the number of evaluated pregnancies:** 

Period	Years	Total No.	Died		Dropouts		Evaluated pregnancies	
			No.	%	No.	%	No.	%
I.*	1960 – 1979	209	14	6.7	17	8.1	178	85.2
II**.	1980 – 1984	276	3	1.1	38	13.8	235	85.1
III.	1985 – 1993	559	2	0.4	44	7.9	513	91.8
Total	1960 - 1993	1,044	19	1.8	99	9.5	926	88.7

* based on the review of admission and medical files of female patients in 1980 admitted to the study hospital between 1960 and 1979.^29,30^ **based on the continuous ascertainment of pregnant women by a psychologist.^31^ ***based on the ascertainment of pregnant women by a sensitive pregnancy test in the blood samples of all admitted women from 15 years to 50 years.^32^

**Socio-demographic status of self-poisoned pregnant women**

The age distribution of self-poisoned pregnant women is shown in Table 2. The maximum number of suicide attempts in pregnant women occurred in the age group from 20 to 24 years. The second most frequent maternal age group was the youngest one (19 years of age or less). However, in fact the maximum was between 18 and 20 years. It is worth mentioning that 5 girls were 14 years old and altogether 77 (8.3%) pregnant women who attempted suicide with drugs were less than 18 years old.

**Table 2: The age of pregnant women at the time of suicide attempts and their proportion according to age groups:** 

Age (yr)/groups	No.	%	No.	%
14	5	0.5		
15	12	1.3		
16	21	2.3		
17	39	4.2		
18	68	7.3		
19	70	7.6		
-19			215	23.2
20	76	8.2		
21	60	6.5		
22	47	5.1		
23	49	5.3		
24	57	6.2		
20 – 24			289	31.2
25	51	5.5		
26	45	4.9		
27	39	4.2		
28	23	2.5		
29	27	2.9		
25 – 29			185	20.0
30	30	3.2		
31	26	2.8		
32	26	2.8		
33	14	1.5		
34	21	2.3		
30 – 34			117	12.6
35	26	2.8		
36	11	1.2		
37	18	1.9		
38	14	1.5		
39	16	1.7		
35 – 39			85	9.2
40	14	1.5		
41	9	1.0		
42	6	0.6		
43	2	0.2		
44	4*	0.4		
40 – 44			35	3.8
Total	926	100.0	926	100.0

* One woman was 46, another one 47 years old

Socioeconomic status of self-poisoned pregnant women was analyzed on the basis of their employment, educational and marital status.

Employment status of self-poisoned pregnant women was recorded in their medical files in the study hospital and was known for all pregnant women(Table 3). There was a predominance of self-poisoned pregnant women with low employment status, compared with the Hungarian pregnant women as a whole (p<0.0001).

**Table 3:Distribution of employment status of pregnant women with suicide attempt:** 

Employment category / status	No.	%	Hungarian pregnant women %
High			
Professional	21	2.0	16
Managerial	138	13.2	22
Medium			
Skilled workers	204	19.5	31
Low			
Semiskilled incl. students and housewives	525	50.3	19
Unskilled incl. unemployment	156	14.9	12
Total	1,044	99.9	100

The evaluation of educational level was based on the number of school years attended in the Hungarian educational system: primary school (including 8 years) is obligatory; secondary school (in general finishing with a final examination after the 12th year), and higher education (with 13 or more years). However, many young females have not finished their school yet. The number of school classes: less than 8 (18.9%), 8 (53.0%), 9-11 (16.9%), 12 (10.4%) and 13 or more (0.8%) was much lower than in the Hungarian pregnant population (p<0.0001).

Socioeconomic status also was evaluated in terms of marital status at the time of self-poisoning. The proportion of married (45.0% vs. 90%), partner (6.4% vs. 3%) and single/divorced/widowed (48.6% vs. 7%) showed significant differences between self-poisoned pregnant women and the Hungarian pregnant population as a whole (these estimations are shown in the bracket as the second figures) (p<0.0001).

The birth order of pregnant women who attempted suicide showed that the majority of self-poisoned pregnant women had their first pregnancy (62.0%), thus they were primiparous (i.e. women with first pregnancy), as the result of their young ages. For 25.8% of self-poisoned pregnant women, it was their second pregnancy and the remaining 113 women (12.2%) had had 2 or more pregnancies previously. However, several self-poisoned pregnant women later had further pregnancies (e.g., of 97 self-poisoned pregnant women who delivered live-born babies in the first period of the project, 79 further babies had been delivered by 1981). ^29^

Thus most women who attempted suicide during pregnancy were young and primiparous with lower socioeconomic status.

**Life-style of self-poisoned pregnant women**

Pregnant women who attempted suicide were in general healthy on the basis of data obtained by a structured questionnaire though personal interview regarding their previous and present somatic and mental diseases. Diagnosable conditions such as depression and panic disorder occurred only in 17 women, this 1.8% rate is not high. However, of 926 women, 30 (3.2%) had attempted suicide previously as well.

Among life-style factors, smoking, drinking and drug addiction during the study pregnancy were differentiated, these data were obtained from the mothers in our institute or at their home visit, sometimes completed with the data of other family members (Table 4). The proportion of smokers was 2.4-fold larger in the self-poisoned mothers than in the Hungarian pregnant population (p<0.01). There was also an obvious difference in the distribution of light (1-10 cigarettes per day), moderate (11-20 cigarettes/day) and heavy (21-40 cigarettes/day) smokers between the self-poisoned and the Hungarian pregnant women, because the proportion of heavy smokers was remarkably higher in the self-poisoned pregnant women. The proportion of drinkers was 14.1-fold higher in self-poisoned pregnant women than in the mothers of the Hungarian newborn population (p<0.001). However, these figures in self-poisoned pregnant women are not correct because most women were drinkers before the suicide attempt (and it was recorded) but not in general. Although relatively few self-poisoned pregnant women were drug addicts, their incidence was much higher than in the Hungarian pregnant population.

**Table 4:Lifestyle factors of 926 self-poisoned pregnant women:** 

	No.	%	Hungarian pregnant women %
Smokers (cigarettes/day)			
1–10	87	9.4	7.6
11–20	209	22.6	11.1
21–40	131	14.1	0.2
Total	427	46.1	18.9
Drinker			
Regular*	135	14.6	1.4
Hard**	74	8.0	0.2
Total	209	22.5	1.6
Drug addict	10	1.1	0.1

*from one drink per day to one drink per week ** more than one drink per day

Thus there was a high proportion of smoking and drinking habits among pregnant women who attempted suicide.

**The time of suicide attempt during pregnancy**

Table 5shows the distribution of suicide attempts according to pregnancy month.

**Table 5:Distribution of suicide attempts during pregnancy according to pregnancy month in the three periods of the project:** 

Pregnancy month	Period I.(1960 – 1979)		Period II.(1980 – 1984)		Period III.(1984 – 1993)	
	No.	%	No.	%	No.	%
I.	0	0.0	0	0.0	213**	37.9**
II.	34*	16.3*	46*	16.7*	128	22.8
III.	50	23.9	76	27.5	71	12.6
IV.	41	19.6	66	23.9	40	7.1
V.	23	11.0	38	13.8	30	5.3
VI.	14	6.7	17	6.2	28	5.0
VII.	21	10.1	19	6.9	23***	4.1
VIII.	12	5.7	9	3.3	22***	3.9
IX.	10	4.8	4	1.4	5***	0.9
X.	4	1.9	1	0.4	2	0.4
Total	209	100.0	276	100.1	562	100.0

* all after 6th pregnancy week ** in the 4th pregnancy week *** one pregnant woman repeated suicide during the same study pregnancy in these pregnancy months

There was a drastic difference in the onset and maximum of suicide attempts during pregnancy in the three periods of the project. The onset of suicide attempts was in the 2nd pregnancy month, while the maximum number of suicide attempts occurred in 3rd pregnancy month followed by the 4th and 2nd pregnancy months in the first and second periods of the project. The analysis of pregnancy weeks within the 2nd pregnancy month showed that all pregnancies were diagnosed after the second missed menstrual period, i.e. 6th pregnancy week by gynecological examination. However, the onset and maximum number of suicide attempts was in the first pregnancy month, more exactly in the 4th post-conceptional week followed by 2nd and 3rd pregnancy month in the third period of the project. There was a conspicuous reduction in suicide attempts during the last two pregnancy months.

Attempted suicides during pregnancy show a very characteristic time pattern. Previous studies showed a peak in the third gestational month,^19^and this finding was confirmed in the first and second periods of our project. Previously pregnancy was diagnosed by manual gynecological examination after the second missed menstrual period; however, the newer, more sensitive pregnancy test can identify pregnancies during or immediately after the first missed menstrual period. Thus, an earlier peak of pregnancy diagnoses occurred immediately after recognition of pregnancy in the third period of the project, when a very sensitive pregnancy test was used.

Nearly all pregnancies were unplanned. Our study design included the clarification of the reason for self-poisoning through a personal interview.^29-32^The most frequent reported reason was that this unexpected event in the life of females was associated with serious conflict with their male partners and/or parents. Twenty-three pregnant women reported trauma during the study pregnancy, all but one woman were beaten by their male partner. The second more frequent explanation for the suicide attempt was economical problems due to their low socioeconomic status. Some pregnant women attempted self-induced abortion by large doses of drugs but they were considered as would-be suicides. Only 3 pregnant women stated that the cause of self-poisoning was unwanted overdose or mixed-up of drugs.

Thus the critical time for suicide attempts was the recognition of unplanned/unwanted pregnancies with a major reason of the conflicts with male partners and/or parents.

**Suicide behavior of pregnant women**

The improving survival rate of pregnant women after suicide is a good news which can be explained mainly by the result of more effective medical care and treatment, however, as a secondary factor, the lower dose of drugs used for suicide by pregnant women is also noteworthy.

Our experiences showed some differences in the suicide attempts of pregnant and non-pregnant women. In general nearly all women used drugs for suicide which were available at home (i.e. used by these women, though sometimes these drugs were used by their parents, mainly their mothers). The first difference between pregnant and non-pregnant women can be explained by this factor. In Hungary medical doctors suggest and frequently prescribe drugs connected with pregnancy complications such as threatened abortion and pregnancy supplements (these medicinal products were recorded in their prenatal maternity logbooks), thus these medicinal products are at home. The symptoms of pregnant women with threatened abortion and preterm delivery are treated in Hungary mainly by diazepam,^33^and promethazine,^34^and this explains why these drugs were used most frequently for suicide during pregnancy. However, 4 pregnant women committed suicide with folic acid as well.^35^The second difference between pregnant and non-pregnant women is that in general pregnant women used somewhat lower doses of drugs for suicide.

Table 6shows as an example the doses of diazepam used for suicide attempts by 112 pregnant women^33^and 112 aged (year) matched non-pregnant females. Both the mean dose of diazepam and the distribution of three dose groups indicated significant differences between the two study groups (p<0.005).

**Table 6: The number of pregnant and non-pregnant women with same age (year) according to the dose of diazepam used for suicide attempt:** 

Dose (gram)	Pregnant women No.	Non-pregnant women No.
0.020	1	0
0.025	5	0
0.030	3	0
0.035	0	0
0.040	2	0
0.050	10	4
0.055	1 34 (30.4%)	0 9 (8.0%)
0.060	1	0
0.065	3	0
0.070	0	1
0.075	3	2
0.080	1	0
0.090	3	1
0.095	1	1
0.100	49	51
0.110	0	0
0.120	0	0
0.125	1	3
0.130	1	3
0.140	0 74 (66.1%)	1 86 (76.8%)
0.150	9	11
0.160	0	0
0.170	1	0
0.200	4	6
0.250	1	0
0.300	7	11
0.350	1	0
0.400	0	2
0.500	2	6
0.550	0	2
0.600	0 4 (3.6%)	2 17(15.2%)
0.650	1	0
0.700	0	2
0.750	0	1
0.800	1	2
Total	112 (100.0%)	112 (100.0%)
Mean ± S.D.	129 ± 752	202 ± 1418

Finally the data of psychological exploration in pregnant women with suicide attempt showed that their psychological crisis was associated with a threatening attitude from her partner and parents.

We may conclude that the recognition of the pregnancy may have some protective emotion for women with suicide attempt. This phenomenon is more obvious after the visible pregnancy, and particularly during the last month of pregnancy. The lower dose of drugs used for self-poisoning in pregnant women may explain their lower mortality.

**Pregnancy outcomes**

The evaluation of pregnancy outcomes showed that the termination of pregnancy due to social reason in these unwanted pregnancies occurred frequently which was supported by the anxiety of the suspected teratogenic risk of drugs in the fetuses.

The aim of Table 7is to show the distribution of different groups of fetal death in the project. The Hungarian baseline figures were known during the study period. The observed rate of miscarriages in self-poisoned pregnant women was lower than expected on the basis of the Hungarian baseline figure. We can suggest only one possible explanation for this unexpected finding: a high rate of early termination of pregnancy after suicide attempt. (The pregnancy outcome was identified in all evaluated pregnant women, thus under-ascertainment is not likely. We disregard only dropouts but these women were deleted from the denominator as well.) The rate of stillbirth was somewhat higher in the first period of the project; however, this group of fetal death did not occur in the third period of the project. It is understandable because suicide attempts were very rare in the last 2 months of pregnancy.

**Table 7: Pregnancy outcomes in the three periods of the project and in the Hungarian pregnant population:** 

Pregnancy outcomes	Period I.(1960 – 1979)		Period II.(1980 – 1984)		Period III.(1984 – 1993)		Hungarian baselines%
	No.	%	No.	%	No.	%	
Termination of pregnancy	68	-	82	-	203	-	-
Fetal death							
Chemical pregnancy	0	0.0	0	0.0	114	(86.4)	NA
Miscarriages (spontaneous abortion)	8	7.3	16	10.5	18	9.2	13
Ectopic pregnancy	1	0.9	0	0.0	0	0.0	1
Stillbirth (late fetal death)	4	3.6	1	0.7	0	0.0	1
Subtotal	13	11.8	17	11.1	132	9.2	15
Live birth	97	-	136	-	178	-	-
Total	178	-	235	-	513	-	-

NA = not available

The major finding of the study is the extremely high rate of chemical pregnancy (positive pregnancy test without any later clinical symptoms of pregnancy) after the suicide attempt in the 4th pregnancy week (of 126 pregnancies which ended in fetal death and live birth, 111, i.e. 88.1% had this diagnosis). Among 24 evaluated pregnancies after the suicide attempt during the 2nd pregnancy month (without termination of pregnancy and dropouts), 3 were diagnosed as chemical pregnancy.

Although the incidence of early fetal loss of pregnancy is very high in general,^36,37^our results have shown that it is particularly high in the earliest period of pregnant women after suicide attempt.

The major aim of our project has been to use the self-poisoning model during pregnancy for evaluation of the potential teratogenicity/fetotoxicity of drugs. Many drugs are subject to contraindications or special warnings because their effects have not been sufficiently studied during pregnancy or non-clinical studies revealed adverse teratogenic or fetotoxic effects^38,39^Data from self-poisoned pregnant women provide an appropriate source of information for a better estimation of the potential human risks of exposure to drugs during pregnancy.^5,40,41^Of course, the possible effects of various poisoning antidotes have also been considered; however, no evidence was found for their teratogenicity in the few case reports available.^42^

Many unwanted pregnancies were terminated after the suicide attempt, and there was an extremely high fetal loss in very early pregnancy, thus only 44% of self-poisoned pregnant women delivered their babies.

**The strengths and weaknesses of the self-poisoning pregnant women model**

Strengths of the self-poisoning model include: 1) Some similarity to animal investigations, in which large doses are administered once on a specific gestational day or during a relatively short period of gestation. 2) The self-poisoned pregnant women are hospitalized, providing medically recorded prospective exposure data for estimating the potential teratogenicity/fetotoxicity of the drug studied in humans. 3) If there is no teratogenic and/or fetotoxic effect of very large doses of the given drug used for suicide during the critical period of fetal development, this information is a very important argument against the human teratogenic/fetotoxic effect of the drug. 4) Dose-response relationships can be evaluated; it is an important criterion of teratogenicity in experimental teratology but not often studied in the clinical environment. 5) The use of the daily clinical dose of a drug in 100 pregnant women is not equal to 1 pregnant woman with a 100-fold higher dose of this drug, due to the well-known threshold effect of teratogenic drugs.

Of course, the limitations of the self-poisoning model are also obvious: 

1) The number of pregnant women who attempt suicide in the critical period of organogenesis (ie, the well-defined sensitive period of fetal development when specific congenital abnormalities can be induced) is relatively low.

2) Most pregnancies are terminated after attempted suicide in early pregnancy, precluding evaluation of defects in fetuses from these pregnancies.

3) More than one drug is often used for self-poisoning, and it is not easy to separate their effects. However, this might be done by use of the appropriate statistical methods.

4) Many drugs are never or only very rarely used for self-poisoning.

5) Although it would be ideal to estimate the effects of the standard treatment dose of a drug, we are only studying the dose used in excess as an overdose in this model. In addition drug effects and the blood level of drugs depend on the time interval between intake and medical treatment and on the efficacy of the medical intervention. However, clinical symptoms of self-poisoned patients may aid in estimating the severity of intoxication.

6) Pregnant women who attempted suicide are younger and had a lower socioeconomic status; therefore, they cannot represent the pregnant population as a whole.

7) Several pregnant women who attempted suicide smoked cigarettes, or consumed alcohol, and self-poisoning itself is frequently combined with alcohol abuse.

8) It is extremely difficult to find appropriate controls for these very specific high risk pregnant women and for their exposed children, although our experiences regarding the use of sibling controls are good.

In conclusion, our Hungarian self-poisoning project among pregnant women has shown that evaluation of drug intoxication during pregnancy due to attempted suicide is an appropriate tool for the evaluation of teratogenic/fetotoxic effects of large doses of drugs and other chemicals.

**Recommendations**

Fortunately the number of pregnant women who attempt suicide during pregnancy is rare, particularly during the critical period of congenital abnormalities. Thus only an international collaboration could provide for a higher number of self-poisoned pregnant women who used specific drugs so that appropriate statistical power could be achieved.

In my opinion therefore it would be worth establishing an international monitoring system of self-poisoned pregnant women (e.g., under the umbrella of the EMEA in Europe or of the CDC in USA or the WHO in the world), based on the following arguments:

1) All self-poisoned pregnant women need special medical-toxicological care in specialized inpatient clinics, and these pregnant women are cared for in a limited number of specialized hospitals (Budapest and its surroundings, including 3 million people, had one hospital for these patients). Thus, their ascertainment is not difficult.

2) Self-poisoned patients are thoroughly examined. Thus, drug concentrations in blood, symptoms and treatments are medically recorded, making the most important prospective data available.

3) The high rate of termination of pregnancy after self-poisoning during pregnancy is the weakness of this approach but most embryos would be appropriate for embryo-pathological examination using the so-called abortion pills (misepristone + misoprostol).

4) Quantitative estimates of exposures can be judged based on the level of drugs in the blood, the severity of clinical symptoms, etc., providing an opportunity to estimate a dose-response relationship, possibly allowing identification of the teratogenic threshold dose of a drug.

5) The self-poisoning model may provide improved knowledge that may aid in better estimating the risk of drugs producing specific defects. Well-directed ultrasound scanning in the second trimester could then be used to identify, or exclude these defects after the use of given drugs during pregnancy, and to protect the life of fetuses without defect, instead of the present frequent termination of pregnancy.

6) Self-poisoned pregnant women at high risk need special medical care regarding the study pregnancy and their further life. Special socio-medical assistance would also be needed to help for their high risk children. An international monitoring system could contribute to solve this socio-medical task.

7) An additional goal is to obtain data regarding the general health, in addition mental and behavioral development of the exposed children, to understand better the human fetotoxic and neurotoxic effects of drugs.^31^

8) Evaluation of the offspring of exposed children or their stored DNA samples provides a special opportunity to estimate reproductive fitness in the third generation ^43,44^, similar to the non-clinical reproductive studies in animals, resulting in a unique approach for evaluation of potential human mutagenic effect of drugs.

9) Finally this international monitoring system of self-poisoned pregnant women may stimulate and contribute to the research and prevention of suicidal behavior.^45^

Thus the final conclusion is that the self-poisoning model of pregnant women within the disaster epidemiology is feasible for the estimation of human teratogenic/fetotoxic risk of drugs and other chemicals. In addition to this scientific benefit, the better medical and social care of self-poisoned pregnant women and their fetuses/children would be important from humanistic aspect.
